# A phenomenological study on the lived experiences of families of ICU patients, Addis Ababa, Ethiopia

**DOI:** 10.1371/journal.pone.0244073

**Published:** 2020-12-18

**Authors:** Habtamu Kehali, Yemane Berhane, Addisu Gize

**Affiliations:** 1 St. Paul’s Hospital Millennium Medical College, Addis Ababa, Ethiopia; 2 Addis Continental Institute of Public Health, Addis Ababa, Ethiopia; University of Edinburgh, UNITED KINGDOM

## Abstract

**Background:**

Family-centered care of ICU patients is increasingly recommended as it is believed to have effect on family members’ psychosocial status and patient outcomes. Defining the nature and extent of families’ involvement in a given health care environment for different stakeholders is a challenge. Understanding the lived experiences of families of ICU patients would help strategize on how to better engage family members for improved ICU care processes and outcomes.

**Objectives:**

The aim of this study is to explore the lived experiences of families of patients in the ICUs of hospitals in Addis Ababa, Ethiopia.

**Methods:**

The study adopted a qualitative approach and a phenomenological research design. In-depth interviews were conducted with twelve (12) family members who were purposively sampled from two government hospitals and four private hospitals. Thematic approach with the application of hermeneutic circle of interpretation was applied to understand the meanings of their experiences.

**Results:**

The study revealed the following major themes: financial burden, challenge in decision making, shattered family integrity and expectations, information and communication gap between family members and health professionals, lack of confidence in the service delivery of hospitals, social pressure against patient families, and families being immersed in an unfriendly environment. Though they do not explicitly mention it to the health care tram, further interpretation of the main themes elucidated that family’s need the intensive care process be cut shorter irrespective of the outcome of the patient condition.

**Conclusion:**

The study gave an insight on the multiple and interrelated challenges faced by families of ICU patients admitted in the hospitals of Addis Ababa. Further contextualized interpretation of their experiences revealed that families were somehow in a state of despair and they implicitly need the ICU care for their family member be ended irrespective of the potential clinical consequences on the patient. The philosophy of family-centered care be advocated in hospitals. The study result affirms the need to include family members during nursing assessment of patients in ICUs and also offers the basis for guidelines development on informational support to the families of the patients hospitalized in ICUs.

## Introduction

The very low critical care capacities and the more risk factors make critical illness more prevalent in developing countries [1, 2]. A larger number of critical patients in these countries are with potentially treatable disease conditions [3]. The incompetent public health action in Africa is accountable for the high burden of critical illnesses from malnutrition, motor vehicle accidents, infectious diseases such as AIDS, malaria, tetanus, etc. [4].

Critical illness is a burden and its measurement is a challenge in the low resource settings [5]. The measurement and early detection of syndromes like sepsis and multi-organ failure often times require a facility with diagnosing devices and skilled clinicians as well. For those critical illnesses that come with early warnings, developing countries usually have a challenge to put in place the very different kinds of early warning detection systems such as early warning scores, algorithms and protocols [3, 5]. As a result, most of the critical illnesses in these low resource countries occur in the form of emergency which will then pose significant challenges on the psychosocial status of the unprepared patient families [3, 6].

Though it is known that critical care in Sub Saharan Africa like Ethiopia is in its infancy stage, there are multiple compelling reasons to bring it in to the public health agenda [3, 7]. The infrastructure (facility), the health care team (health professionals), the patient and the family members of the patient are the integral parts of a health service delivery in hospitals [8].

Intensive care unit (ICU) is one of the wards in hospitals where critical care service is given. Families of ICU patients are the source of information including past medical history of the patient [8]. They also do shared decision makings, pay the medical bills, function as a legal representation to give consent and respect patient rights, and do the routine care for the other affected family members of the patient at home [2, 8]. Families are often times overwhelmed with a lot of the above stated activities and therefore may experience different issues and challenges such as psychosocial trauma and/or crisis within the family [6, 9].

Literatures on families of ICU patients identified many health related challenges and issues such as lack of communication among family members, feelings of overriding threat including vulnerability and uncertainty, intense emotions, disruption of home routines, poor nutritional intake, changes in relationships, role conflict, and physical illnesses especially among patients' children [6, 10, 11].

There is a growing evidence that mutually beneficial partnership among families, patients and health care providers called ‘patient-family-centered care’ improves outcomes for both patients and family members [6, 12]. The involvement of families in critical care, besides its impact on the health prognosis of the patient, has shown to reduce the psychosocial stresses and crisis of families as a result of the illness of their loved ones [6, 8] and also increase the families’ satisfaction with the critical care which in turn has effect on the rate of health care utilization [12, 13].

Despite the advocacies of policy makers and researchers on the importance of patient-family-centered care, there is no clear consensus among stakeholders on the nature and extent of the patient and family involvement during the care delivery [6]. Family ‘engagements’ is also a complex concept beyond the mere interplay between family members, the healthcare and the social environment [11, 14–17]. Understanding how families make sense of their experience in the hospital is instrumental to develop hospital based guidelines and protocols in order to implement the family-centered care [9, 11, 18]. The researcher also couldn’t find any official guideline drafted at the national level in Ethiopia regarding the engagement of family members in the hospitals’ ICU care.

The experiences of families of critical patients are individualized and most importantly dependent on the setting of the intensive care facility [9, 11]. Many of the researches done in the ICU settings of developing countries are concentrated on patients’ biological and psychological well-being or family’s’ needs and satisfactions which mostly are quantitative [3, 7, 19–22]. Despite the extensive search, we found limited evidence in Sub Saharan Africa including Ethiopia regarding the families’ lived experiences in the hospitals ICU environment.

In Ethiopia, there is a requirement for the health care staffs in ICUs to inform and take consent from patients or families during admissions, prior to initiating or discontinuing critical procedures and care plans. Healthcare service fee is mostly out-of-pocket payments. User fees are usually higher in private settings but highly subsidized to make it affordable in the government hospitals. There is a wide range of variations of ICU facility amenities and visitation rules among hospitals. Empirically and relatively, private hospitals are run with smaller ICU capacities but better amenities for patients and family members, and have cleaner ICU environments compared to the government hospitals. Hospitals tend to have teams, committee, or decision boards to deal with varied ethical, legal, and quality related issues.

The researcher believes exploring the families’ experience in the ICU settings gives a better understanding of the nature of interactions between families and ICU environments with in the context of hospitals in Addis Ababa. The researcher pursued the study wondering what it is like to be a family of a patient admitted in an ICU of hospitals in Addis Ababa.

## Methods

### Study area

Currently, according to the Ethiopian Health Directory printed in January 2015, there are 11 governments and 35 private hospitals in Addis Ababa city. More than half of these hospitals have ICU facility of single or mixed specialty. Six hospitals were included in this study: St. Paul ‘s Hospital Millennium Medical College, Ras Desta Damtew Hospital, Bethel Teaching General Hospital, Yerer General Hospital, St. Yared General Hospital, and MCM Hospital. The first two are public hospitals; MCM is a missionary hospital; and the other three are privately owned. The ICU capacity for the hospitals during the time of this study ranged from 4 beds to 12 beds.

### Study design

Phenomenological study using in depth-interview was conducted to bring a relatively new insight for the context and to capture the meanings made by participants. The primary researcher, as a clinician who during the study period had been working in ICUs of hospitals in Ethiopia, has pre-understandings and /or biases and judgments regarding the phenomenon under study. Because the researcher is neither a stranger for the phenomenon nor able to completely suspend (bracket out) all the prejudices and pre-understandings about the phenomenon, the Husserl transcendental type of phenomenology was not the choice [23]. According to Gadamer (1976), it may not be possible to fully ‘eliminate’ one’s own view point regarding a phenomenon called ‘horizon’ which is again influenced by the researcher’s prior belief, experience, professional background and act of seeing things [24]. The Heidegger hermeneutic style of interpretation however allows the researcher to bring those horizons as a ‘lenses’ to describe, explain and interpret for the meanings of the families’ lived experiences [24].

### Study population

The source population was all family members of patients admitted in the hospitals’ ICU in Addis Ababa. The actual study population was families (family members, relatives, next of kin, close friends, guardians, primary care takers, etc) who identified themselves as the ‘family’ of the patient admitted in the ICU for at least two days, and also designated and /or documented as a primary care taker of the patient by the head nurse or the treating physician.

### Inclusion and exclusion criteria

An inclusion criterion was a family member and a designated primary care giver who is at least 18 years old and cooperative to go through in-depth interview. Even if designated as a primary care giver, family members with any one of the following criteria were excluded from the interview: a family of a dying patient, a family age less than 18 years old, a family apparently in distress during approach by the researcher, and a family of a patient who stayed in the ICU for less than 48 hours. In order to deepen understanding and as part of an endeavor to bring varied ‘horizons’, the researcher purposefully sought participants from multiple hospitals both from private and government ones.

Critical care unit and Intensive care units are interchangeable for this study. Critically sick patient is a patient admitted in an intensive care unit (ICU) of a hospital and who is not in ICU only for monitoring purpose in anticipation of a life threatening disease condition [9, 10]. To be considered a critically sick one, the patient should be placed on at least one medical intervention. Family member is an individual that is related with the patient by bonds of marriage, blood, adoption, or a close friend and acquaintances acting as a primary caregiver or providing family role for the patient in the ICU care. Qualified family member is a family member at least 18 years old who is designated as a primary caregiver by the head nurse or the treating physician of the critically sick patient. If more than two family members are qualified, the investigator will use his discretion to select two family members based on their level of willingness to communicate and their attitude towards the research objective.

### Sample size and sampling

A maximum of two family members per patient and a maximum of 3 participants per hospital were included. A total of 12 qualified participants were interviewed from six of the above listed hospitals. Purposive sampling was used to select key informants also called ‘information-rich cases’ who are assumed to be having a different perspective or information, willingness to talk, and better experience on the subject matter.

Treating physicians or ICU head nurses of the respective hospitals were first informed of the study objective and asked to identify the patients’ duration of stay in the hospital ICU. The investigator then approached the family members in order of the patients' duration of ICU stay, the longest first, and checked if they qualify. The approached family were then given a written information regarding the nature of the study including the purpose and the fact that they have the right to terminate their participation at any point during the study. In the meantime, the principal investigator evaluates if the individual encountered falls under the inclusion criteria. If the longest doesn’t qualify, the researcher then checks the second longest and then the third and so. If and when the participant qualified and willing to participate, they are asked to sign on the consent paper.

### Data collection tools & procedures

Firstly, appropriate permissions to conduct the study were gotten from the respective hospitals administrators (IRB board in the case of St. Paul’s Hospital Millennium Medical College). At the start of the interview, if and when they don’t have anything to ask, they were then asked to sign on the consent form. Using a semi structured interview guide, in-depth interviews were conducted, and possibly audio-taped. In addition, depending on the context, field notes, participant casual encounters, reflective journals were used. Data was collected in the months of February and March 2015 by the principal investigator. Participants were privately interviewed seated at a relatively quiet and comfortable spot or room in the respective hospitals’ compound. The interviews were started with open ended questions: ‘what was it like for you to be a family of a patient admitted in the ICU of this hospital?’ For the four participants who refused to be audio-taped, the principal investigator actively took field notes during the interview.

### Data quality

Participants' identification code, which contains the source hospital and interviewee specific alphabet code was created and written on the consent form right after the consent is signed (confidentiality). Participants at the start of the interview were clearly oriented that the interviewer was not part of the hospital healthcare team and cannot intervene in the patient management, that the information and the result of the study (whatsoever) had no influence on the care of the then ICU patient, and that the researcher role then is only as a researcher and not a health professional one. A semi-structured interview guide was used to help address all the participants with relatively similar questions. In the meantime, the primary investigator was open and alert for any new emerging ideas during the interview. The new emerging idea was made to be open for possible new meanings and interpretations while the researcher remained cognizant of the temporality of the ‘truth and the horizons of the interpreter and the text’ [23].

The researcher, a clinician who had been working in different hospitals, used his prior ICU experiences and understandings of the phenomenon under study to elicit concerns and meanings of the lived experiences being narrated [23, 24]. Interviews were conducted with the authentic search of divergent perspectives and not with an intent to justify the pre-understanding of the researcher. When the researcher believed that a divergent pattern of experience emerged, the summary of the new emerging perspective was repeated to the participant (member checking) which further contributes to the credibility of the study. None of the participants asked for the correction of the briefings nor dropped in the middle of the interview. The engagement with each of the participants was therefore fairly prolonged which helped the researcher to draw rich information from each participant [24].

Soon after the completion of each interview, the investigator did listen the recorded files and/or read the field notes again and again to capture the pressing themes, concepts and perspectives of the interview. Reflective journals along with important quotations from the respective interview were separately noted on the same day of the interview. The primary investigator himself transcribed verbatim and translated the recorded audio files as soon as possible to help recall the very details of the recorded pieces.

### Data analysis

Data analysis was ongoing process beginning with the first interview. Data analysis was performed by applying the hermeneutic cycle that constitutes reading, reflective writing and interpretation [24, 25]. Thematic approach along with abstract interpretation was applied. The primary investigator’s pre-understanding of the subject matter and the reflective journals were utilized to easily grasp the entirety of the data content during analysis. The researcher’s understandings reflected in the journals after each interview were evolving from interview to interview. These temporal ‘truths’ or horizons were again fused with the main themes arising from the transcribed texts. The fusions may have resulted to a divergent concept or remained in the same horizon. Hence, during analysis, there were a back and forth movements from the ‘whole’ meaning or concept to the ‘parts’ of the texts and themes called hermeneutic circle [23, 24]. The researcher finally tried to merge the main emerged themes and developed a hypothetical concept on the experience of families of ICU patients in the Ethiopian context. Open Code Software version 4.0 for qualitative data analysis was also used during the analysis process.

### Ethical considerations

Firstly, ethical approval was obtained from the Internal Review Board of the University of Gondar and ACIPH, and submitted to for each hospital to get a permission. Then, participants were advised in writing about the voluntary nature of the study. Data collection only started after signed informed consent is obtained from the respective participant. Alphabet codes were used to identify the participants instead of their names. The participants were advised that, at any time during the process, they could withdraw or decline to answer any question or discuss about any issue. Collected data from participants such as recorded audio tape cassettes and field notes were retained only at the possession of the primary investigator.

## Results

The study participants were family members of patients who stayed in ICU ranging from less than a week to more than 2 months (Table 1).

**Table 1:** Family member participants’ demographic data.

Participant Code	Age	Gender	Educational Status	Relationship to the patient	Duration of the patient’s ICU stay in weeks*
A	52	M	College	Father	3
B	45	F	High school	Wife	6
C	38	F	College	Wife	4
D	42	M	High School	Father	9
E	40	F	High school	Wife	7
F	38	F	High school	Daughter	6
G	32	F	College	Daughter	3
H	45	F	Elementary	Wife	3
I	50	F	Elementary	Sister	2
J	40	M	High school	Brother-in-law	2
K	34	F	College	Daughter	1
L	38	M	College	Brother	3

*4 or more days are rounded to a full week.

Most of the experiences narrated by the participants were inclined towards the challenges they experienced as a family of a patient admitted in ICUs. The study analysis revealed the following main themes: financial and psychosocial burden, dilemma during decision making and information sharing, shattered family expectations and family integrity, information and communication gap between family members and health care teams, lack of confidence in the hospitals’ service delivery system, families being immersed in an unfriendly environment and a sense of fulfillment in helping the patient. The analysis outcome with the themes and sub-themes are revised below (Table 2).

**Table 2:** Analysis outcome, main themes, and subthemes of issues and challenges of family members of critical patient admitted in Addis Ababa hospitals ICU.

Sub Themes/Codes	Themes/Main Categories	Analysis Outcome/Theories
• Not enough money in pocket during admission	Financial Matters	Families encounter different stressors and live in the world of uncertainty and they want the "ICU care" to end sooner irrespective of the patient outcome.
• Most of the medications or laboratory are bought/done outside from private health facilities		
• The day-to-day expense is very high and is more than expected even in the public hospitals		
• Family caregivers usually go off work unofficially and may lose their job		
• Money matters are not given due attention during decision makings in ICU		
• Whether to continue or withdraw life supporting care	Decision Making	
• Decision whether to accept or decline offers for referral		
• Decision whether to release or hold information about the family or the patient		
• Overwhelmed with many matters requiring immediate decision making		
• Family members don't maintain their support all the way through the course of ICU stay	Expectations & Family Integrity Threatened	
• Home routines such as care for kids is disrupted		
• One family member of the patient may not trust the other		
• Attention and support will be directed only to the critical patient		
• No clear information about the patient condition	Information Gap	
• No uniform information or understanding between the family members		
• Not sure if the doctor or nurses are having the right & all information about the families or the patient		
• Families may not be sure if the patient is having the available good care	Lack of Confidence	
• Families may not trust nurses if the prescribed drugs and/ or food is being given to the patient		
• Orders and prescriptions may sometimes be not in favor of the patient but the hospital revenue		
• Not confident with the idea of oneself or other family member		
• Working hard only to fulfill social expectations	Psychosocial Pressure	
• Trying to sneak into ICU and do religious practices without being noticed by the health care staffs in ICU		
• Overwhelmed by visitors		
• Loss of ideas when family members come face to face with doctors		
• I have done my best	Sense of readiness to accept	
• There is nothing to regret whatsoever is the outcome		
• Good feeling on what they have gone through to support the patient		
• Lack of privacy with oneself, patient or family members	Unfriendly environment	
• No place to sit, pray, do family gathering or rest		
• Poor toilet sanitation		
• The ICU room design take the patient away from family members view		
• Cafeteria is not 24 hours open		
• The building is new and confusing; family members face new doctors or nurses every frequently		

### Financial burden

Financial and psychosocial burden were the main issues families emphasized during the interview encounters. Participants said the following:

*Oh the cost is so high*, *my brother*!?…*private is unthinkable*. *Here the laboratory and the drugs are almost always from the outside*… .*Even to take a single sample for lab to the outside and the result back to the hospital is something hard financially*, *time taking and tests our patience*..*(H)*

*I am now worried for my child may be referred to another governmental hospital*. *The guy who caused the accident claimed he doesn't have any more money to pay and wants my son go to Black Lion Hospital or somewhere in the government hospital*, *I know he pays less there*.,, *I however want him stay here as the care is good*..*(B)*

One participant emphasized the medical service costs even in public hospitals are not affordable due to the fact that medications and laboratory services prescribed for patients are not always available in public hospitals.

*We on average spend from 300 to 400 birr per day to purchase medications from the outside private pharmacies*…*imagine the cost of a ICU room for a patient is only 2*:*50 birr per day in this hospital (St*. *Paul’s Hospital)*, *and yet you just pay that amount for a single medication just because it is not in the hospital's pharmacy*..*(It is)better to increase the bed rent and make those expensive drugs available in the hospital*…*(G)*

Financial challenge is highly inter-connected with other elicited themes. For instance, one participant whose husband was admitted in a private hospital expressed in despair how the high cost of the ICU care threatened her family integrity and created a difficulty in decision making weather to continue care for her husband or not:

*I myself don't want to decide but I want the discontinuation of the care*,,, *I know it is futile exercise and I don't have any more birr (money) to pay for the expenses*. *I wish the decision comes from the doctor and care be discontinued right away*. *Or else our son will hate me later as if I caused his death only to save money*,,, *he will think that way ever*…*(K)*

Even if one can afford to pay, there are circumstances that families need money and yet don't have the convenience to access cash. The problem of cash accessibility comes in to play especially during emergency admissions as commented below by two of the participants.

*How can one carry a 10*,*000*.*00 or more birr during such emergency circumstances; I was told to deposit such money right away in advance for him to be admitted in ICU*.. *Life should have come first before money things*… *(D)*

*We experienced problems especially during admission time; my sister who could tell a better history for the doctor about our mom's condition was already out in the city looking for money (ATMs)*. *It was a whole lot of mess*, *I wish the hospital accepts credit cards*, *or hold IDs till we deliver cash*, *or ATM machines need to be around/close by the hospital*…*(E)*

Families, as their deposit is drained paying medical bills, they start to frustrate and tend to lose hope in the final outcome of the patient.

*If my husband is going to die*, *why did they initially gave us hope and tried all these expensive treatments and supports (K)*

*If money was to buy health*, *our father could have been totally healthy long time ago*..*(D)*

To make things worse, a family caring for a critical patient in ICU could also face legal issues. For instance, it is not socially acceptable to talk about family financial related settlements and legal matters while the patient is critically sick even if it is probable that the patient is going to die anyways.

*I am not able to talk about financial matters of our household with my sick husband*,,, *you know in case he doesn't make it*, *I need to also worry about our daughter's future*. *In the meantime I am afraid of his families*,,,*(she couldn't finish the sentence) (L)*

### Decision making

Families of critical patients can get overwhelmed with relatively "new and complex" issues requiring their immediate decision. Families are requested to give consents urgently in the settings where they are unprepared and not well informed.

*We requested to give us more time to reach a decision but they hurried and told us it was an emergency; in fact*, *the reality was we didn't even know what was happening and we had no good understanding of the real problem*, *late alone to make a good decision*…*(D)*

*They asked me to decide and sign a document urgently; they told me it was emergency*, *I did sign on a paper which I didn't clearly know what it was for*,,, *(D*)

There was a lady who really wanted her dad to be referred to another affordable hospital. In order for this to happen, she normally needs to inform for the doctors and they are the one to communicate and find a receiving hospital. Nevertheless, she was afraid to ask for it because she believed the doctor could lack empathy towards her dad in case the referral is not successful due to many reasons.

*I feel and wanted to take him out of this (expensive) hospital but I am afraid and don't have tangible reason to ask for*…*it will be worse if I mention this (need to change hospital) and they finally couldn't find a hospital to refer my dad to… (D)*

The other challenge stated by multiple participants is the decision revolving around whether to continue or withdraw a life support modalities or expensive medications. Participants indicated that the medical bill from ICU can ruin the future life of the rest of the family members and the ICU care usually is a futile exercise. The situation in ICU is more unpredictable as discussed by a lady who was caring her terminally sick older sister for more than two weeks in ICU:

*Each of them (the doctors and nurses) know she is dying anyways and hence why not continue with the very basic cares and medications*,,, *why waste the family money*,,, *they must understand and think of the rest of family members future life*..*(H)*

*I asked the doctors how much money is enough to really know if she is going to die or survive*, *and they don't know*. *We are in dilemma if we need to keep spending for her medical bills or not*.. *(H)*

### Expectations and family integrity shattered

Families may expect more social and financial support from others during such times. They however seemed to experience the other side of the story through the course of the ICU care. The longer their loved one stay in the hospital, the more their home routine is disrupted and the lesser social support is available. One participant said the following:

*My kids at home are sick of cough and diarrhea*, *may be due to lack of proper care at home*. *Neighbors and cousins usually flock to here just to pay a visit to her*; *they could have helped us in another good way by doing some homework for us*…*(F)*

The other challenge is financial matters which threatened the trust among family members.

*All the money I sent for my mother’s medical expense was partially redirected to his (my brother) own use*. *He could have at least visited our mother even if he can't help financially*, *I am really sorry for the generation*..*(J)*

Often times, part of the family insists for aggressive trial of any intervention to rescue the life of the loved one. The other part of the family, however, might be logical and reserved in spending more considering the future life for the rest of the family. A lady attending her critical husband in ICU said," Our son is not happy with me when I suggested affordable ways of helping his dad; he thinks we have more money to spend for his dad's treatment."

One participant who was frustrated about caring her sick mother with no other support stated the following:

*It was other peoples who brought her to this hospital*, *who currently disappeared*. *When friends*, *neighbors and our son preferred to come to this hospital*, *I couldn't say no*, *or else our son will hate me and think about something else (K)*

### Information and communication

It is known patients' family members need clear, understandable and honest information from the health professionals regarding the condition of the hospitalized patient (19, 27). Participants acknowledged that the treating physicians meet them on a regular basis at least once a day to brief them on the patient condition. Families however lose the nerve in front of the doctor to speak up and discuss what they have in their mind. One participant said, "I don't know but I have a feeling my doctor doesn't know every information I would like him to know." The other one added, "Sometimes I lose my ideas when I face the doctor to discuss about my husband situation (C). "

Families also face information gap among themselves. There could be no uniform information or understanding between members of a given family. Sometimes information could be deliberately held from part of the family members or patients for a seemingly good notion as stated by a daughter of a cancer patient:

*I suffered and worried a lot in hiding the information that mom’s problem is cancer*,,, *the doctors doesn't really understand that she would be broken in a day if she were to learn her actual disease condition*,,, *I even came across with a doctor who threatened me that I am totally wrong and denying the autonomy of my mother*,,,*(E)*

Families struggling to preserve sensitive information about the health condition of their loved ones could face a lot of social pressure, as a result. This was also well described by the participant:

*All my family members don't know the real problem of hers as I do*,, *and therefore they want me to continue the treatment and tells me she will be alright*,,, *they even complained why she is not getting treatment from another affordable heath institution*,,, *they just don't understand the situation*…*(E)*

### Lack of confidence or trust

Families of ICU patients may lose trust to the health care delivery system, to the health professionals, or to their family member(s). One of the participants mentioned that she was not confident if her mother were getting a proper care:

*I sometimes feel sad that the expensive drug I am buying is not all properly given to him or the expensive drug was not in the first place appropriate to be ordered*,,, *(Do) you know they may sometimes order such medications to generate revenue for the hospital*,,, *what can one say then*, *or the doctors does not exactly know the problem of my husband*, *then*? *(K)*

A previous experience of losing a family member in an ICU of a hospital makes the scenario worse. For instance, the principal investigator encountered a frustrated participant who said the following about her mother admitted in ICU:

*My father died a couple of years ago here in Addis Ababa in another hospital*. *I didn't get a chance to say good bye to him at the last moments of his death*. *We missed him by the night we thought he was much better*. *This time therefore I always leave ICU as if I see her the last time*. *What a bad feeling*,,, *cry*,,, *don't know if I will see her again*,,,*(I)*

### A sense of readiness to accept

Being there close to the seriously ill loved one seems to give for the family a sense of readiness to graciously accept the patient outcome. Taking the role of a guardian, sharing decisions on the care plan, being the source of information for the visitors, and covering the cost of the medical bills gives them a sense of no remorse on what was done for the patient despite the patient’s outcome.

*I have really done my best for her and the rest is to God*. *I think the doctors are also doing their best*,,, *I do have a good interaction with them*. *They keep me abreast of any update on her condition*. *I pray for the best and there is nothing I regret it*,,, *(A)*

### Psychosocial pressure

Families were also worried about the lack of support from the other family members and relatives at home. In addition to the stressful hospital environment, families are also bothered and sometimes feel uncomfortable by ‘too many’ visitors.

*When a lot of visitors were allowed to come in*, *we worry about the spread of infection*, *health staffs being interrupted from their work*; *we don't know if she is happy or not to be seen by those people*…*(F)*

*I am tired of informing visitors regarding the patient condition which I myself am not clear with*..*(D)*

*Every time a patient die and families cry in the compound*, *my hope vanishes and I feel like I am the next to learn the death of mine (my father)*,,, *(D)*

One participant revealed that he was caring the patient just to fulfill his social responsibility and expectations from his families.

*Here at the hospital*, *I see and realize how one's own life is easily gone (pass away)*. *I am feeling prone to death*,, *the more I stay the lesser I expect new developments about his health*. *I started simply to take their order and bring the medication and food they want*. *I am no longer eager to see new things*…*(G)*

A participant also experienced some difficulty to comfortably do religious practices on the patient side.

*I assume practicing some spiritual things at the bed side near him makes the nurses and doctors unhappy*,,, *anyways I did some without the awareness of the care givers (nurses and doctors)*, *(C)*

### Unfriendly hospital environment

The common comments raised by almost all the participants is the need for hospitals to have a waiting area (family room), and something like a chair or a couch to rest or lie down during night times at least for a single family member per ICU patient.

*When he (the patient) was in the general wards*, *I used to get something like a chair or couch to sit and to lie down during night times; but now*, *there is nowhere for us to stay around especially night times*..*(G*)

The absence of a room for families to eat, rest, meet, pray, etc is also one of the issues repeatedly mentioned for different hospitals.

*There is no place in the hospital where I can pray*, *comfortably sit*, *eat or do some private talking with my families*. *I am here right outside the door getting panic every time the ICU door opens and a nurse or doctor comes out*,,,*(J)*

Because there was no room dedicated for family members to stay in the hospital, participants themselves complained of some physical illness too.

*I myself has got back pain; I was supposed to go for surgery for this*, *I am taking medication; I don't know why I am feeling worse these days*, *may be due to the cold weather and no proper family room for us*. *You just can't stay at the cafeteria*; *it is expensive*…*(J)*

## Discussion

The study explored the lived experiences of patient families in the settings of hospitals' ICU. Descriptive quotations were preferred as a way of thick description of the families' experience. Also, tacit knowledge gained during the data collection process and the investigator's previous personal experience was applied during content analysis. Generalizability is an issue for qualitative studies and it is not an exception for this study. However, in order to make the result more useful for public health interventions, analytical generalization was made for this study. Hence, the main theme of this study- families undergo through a lot of stressors, live in a world of uncertainty, and want the ICU care process to end sooner irrespective of the patient outcome- is a conceptual analysis outcome of the study.

The major issues and challenges described by family members are very much interrelated; and one could be the effect or cause of the other. For instance, lack of confidence, information gap, psychosocial pressure, and financial problem have significant effect on the decision making process of family members which then may have impact on the overall prognosis of the health of the patient. The investigator believes most of the challenges stated by the participants could be tackled at the level of the interaction of health professionals with family members. Unfriendly environment, however, need to be solved at the level of hospital management while financial strain of the family members has something to be dealt more at the level of policy makers.

The literatures reviewed on issues and challenges of families of a critical patient documented similar themes with the findings of this study [26–29]. The question could be why it is similar despite totally different settings. The investigator believes the themes of studies conducted on challenges and issues of families (the stressors) at different contexts are only similar at face value. The depth, the breadth and the root cause of a stressor however is different from context to context. For instance, a study done in United States by Auerbach et al on satisfaction with needs met of patients' family members pointed out that the "alien hospital environment" is one of the challenges for families of ICU patients [26]. He described alien or unfriendly environment as new building facility and new people with a totally new set of rules and regulations. In this study however, unfriendly environment refers to uncleanness and lack of basic facilities in the hospitals.

High cost of medical bill as a challenge for families of critical patient is also well stated by literatures from both developed and developing countries [15–17]. What is interesting from this study is families usually consider their expense in ICU care as part of a futile exercise, and they do it just to take the course of the care to a final point and to fulfill the social expectations. Even, many wish the ICU care process for their family member ends sooner as they think it brings as such no effect on the final outcome of the health of the patient they care.

Regarding the environment of the facility, the investigator believes families are not asking too much for our context. It is just a clean toilet, a one common family room in a hospital, and one convertible couch per ICU bed. The absence of these, however, seems to be creating a lot of dissatisfactions from the family side.

When it comes to decision making as one of the challenge faced by families of a critical patient, it is not that families of this study were given clear and understandable options and they fail to choose. Families however were simply put to decide and /or sign consent urgently in no time or with limited information regarding the situation. They are also in dilemma for they think they might have signed against the interest of the patient or even to the physicians. There are studies showing families don't want to decide because of heightened anxiety especially during emergencies [14, 28].

The participants of this study mentioned that communication is one direction from the health professionals to the surrogates and only clinical factors are discussed as part of 'informed' consent process. Patient families are shy during critical decision makings, and therefore families' ability to pay the cost of the medical procedure or drug is not given due attention during decision making. Studies done in Black Lion hospital revealed the deficient patient-doctor interactions (family-doctor interaction in the case of critical patients) in our context [7]. And, unfortunately, empathy shown and good patient-doctor interaction are found out to be one of the key determinants for patient (families) satisfaction [15, 26, 30].

Incongruent with other studies, participants of this study across the board mentioned that they should not be denied of frequent and clear information about the status of the patient [15, 18, 22, 31]. All the hospitals used for this study has strict regulation regarding family visit to their ICU patient, and interestingly all the respective families liked the limited visitation rule of their hospital.

It is known that reality is also time bounded. Which of the main themes of issues and challenges of families comes at what time line of the patient ICU stay is also an important point analyzed in this study. At the early stage of ICU stay i.e. from admission till they stabilize, families need more information and need more proximity to the patient than later times. Also the issue of access to cash from their personal account comes to be an issue during and early after admission. As their stay in ICU is prolonged, obviously the issue of medical bill along with the dilemma in decision making surfaces. The more they stay the lesser hope they have on the health outcome of the patient but better satisfaction with the health care delivery. The satisfaction may be due to better interaction with the staffs and adaptation to the hospital environment as they go along.

The study has strengths in terms of methods and result analysis. All the study participants were interviewed during the time of the patient's hospitalization in ICUs; the issue of recall bias is almost eliminated when participant families describe their lived experiences. Investigator's previous experience in ICU environments was useful to easily grasp the content of the interview data. In order to get more ranges of perspectives and experiences from families, the participants were drawn from all types of patient-family relationships, from both private and public hospitals and ranged from recently admitted critical patients to the ones with a prolonged stay in hospital ICU.

The study, however, has the following limitations: (a) The investigator was not sure if data saturation was really achieved due to time and money constraint; more interviews may have elicited further perspectives, challenges and issues of family members of a critical patient; (b) The primary investigator found it difficult to keep himself distant from the participants (bracketing), the effect of which may compromise the objectivity of the study results which is in fact the challenge of many qualitative studies [11, 23, 24].

## Conclusion

Family members undergo through a wide range of concurrent stressors which may have direct and indirect effect on their personal health, patient's medical outcome, and the family future life. Interaction with family members need to be part of the nursing assessment of the patients in ICUs. Health care team need to bear in mind the emotional, social and financial status of families during ICU patient care plans including when taking consents and shared decisions makings with families.

Not all critical patients are brought to the ICU of a hospital with the aim of salvaging the life of the patient. Some families go through the ICU care process just to fulfill the social expectations. The concept of Informed Consent should not be overlooked by health professionals working in ICU of hospitals in Addis Ababa. The interpretation of the themes pointed to the meaning that families implicitly want the ICU care process to end sooner irrespective of the patient outcome. Further large scale quantitative study need to be done to clearly identify or solidify the issues and challenges of patient families in hospital ICU settings.

## Supporting information

S1 Appendix In-depth interview guide (semi-structured questionnaire). (DOCX)

## Abbreviations

AIDS: Acquired Immuno-Deficiency Syndrome
ICU: Intensive Care Unit
MCM: Myungsung Christian Medical Center
WHO: World Health Organization
