# Supplementary material for: A phenomenological study on the lived experiences of families of ICU patients, Addis Ababa, Ethiopia
Source: PLoS One. 2020 Dec 18;15(12):e0244073. doi: 10.1371/journal.pone.0244073 (PMC7748272; doi:10.1371/journal.pone.0244073)
Supplement: S1 Appendix — (DOCX) [file pone.0244073.s001.docx]

**S1 Appendix:**

**In-Depth Interview Guide (Semi-Structured Questionnaire)**

1. How does it look like to be a family of a patient admitted in the ICU of a hospital?
2. Please tell me what do you feel, think, or makes you worry right now in relation to the fact that your family member is critically sick and why?
3. Could you please help me understand the details of the experiences you as a care giver are now going through? You may narrate me experiences which may be related to:
   1. Cost of medical bill and other expenses
   2. Sharing the decision whether to apply or withdraw a supportive, therapeutic or diagnostic modality for the patient, and giving consent
   3. Dealing with sensitive medical information
   4. Coping up with the emotional or physical distress, if any
   5. Regret about something you think should have been done for the patient
   6. Social pressure around hospital and home
   7. Taking care of the families and close relatives affected by this situation
   8. Significant outsiders; e.g. Health professionals, out of town or out of country relatives, financial supporters, elderly and/or religious peoples, and so on
   9. Health professionals behavior, Confidentiality, Knowledge, and Skill
   10. Things related with referral of the patient, if any
   11. Information from and communication with health professionals
   12. The absence or scarcity of recommended medical equipments
   13. The hospital environment by itself
   14. Issue towards visitation of the patient while in ICU
   15. Trust on confidentiality, the knowledge and/or the skill of the health professionals
4. Do you feel the treating physician knows everything you would like him/her to know about the patient, or everything you think is medically important?
5. Do you feel you are well considered, informed, and /or treated by the health professionals attending the patient?
6. Which challenges and issues are getting easier or worse as time goes on, or as you get along with the process of caring the patient?
